# Insects as Novel Food: A Consumer Attitude Analysis through the Dominance-Based Rough Set Approach

**DOI:** 10.3390/foods9040387

**Published:** 2020-03-27

**Authors:** Rocco Roma, Giovanni Ottomano Palmisano, Annalisa De Boni

**Affiliations:** 1 Department of Agricultural and Environmental Sciences, University of Bari Aldo Moro, 70126 Bari, Italy; rocco.roma@uniba.it; 2 International Center for Advanced Mediterranean Agronomic Studies (CIHEAM Bari), 70010 Valenzano, Italy; ottomano@iamb.it

**Keywords:** entomophagy, consumer analysis, DRSA

## Abstract

In Western societies, the unfamiliarity with insect-based food is a hindrance for consumption and market development. This may depend on neophobia and reactions of disgust, individual characteristics and socio-cultural background, and risk-perceptions for health and production technologies. In addition, in many European countries, the sale of insects for human consumption is still illegal, although European Union (EU) and the European Food Safety Authority (EFSA) are developing regulatory frameworks and environmental and quality standards. This research aims to advance the knowledge on entomophagy, providing insights to improve consumer acceptance in Italy. This is done by carrying out the characterization of a sample of consumers according to their willingness to taste several types of insect-based food and taking into account the connections among the consumers’ features. Thus, the dominance-based rough set approach is applied using the data collected from 310 Italian consumers. This approach provided 206 certain decision rules characterizing the consumers into five groups, showing the consumers’ features determining their specific classification. Although many Italian consumers are willing to accept only insects in the form of feed stuffs or supplements, this choice is a first step towards entomophagy. Conversely, young Italian people are a niche market, but they can play a role in changing trends.

## 1. Introduction

In Western societies the practice of eating insects, also known as entomophagy, is not usual in traditional diets, so that insects are rarely considered as edible [1]. However, insects can become a possible alternative to animal protein source thanks to their richness in protein, fat, minerals and vitamins [2], lower request of land and water [3], lower environmental impacts in terms of fewer greenhouse gases emissions and ammonia production [4], and also due to their more efficient feed conversion rate with respect to conventional meats [5,6]. In spite of the growing interest towards these benefits and the subsequent debate around the theme of insects as food, most of Western consumers still have reactions of disgust and rejection against them [7]. Generally, the main obstacles for consumers’ acceptance of novel food (defined by EU Commission as “food that had not been consumed to a significant degree by humans in the EU before 15 May 1997”) are food taboos and socio-cultural and psychological barriers, so that the aspect of a food can cause a disgust-based food rejection [8,9]. Indeed, the evident references of a food’s origin to an animal (i.e., its “animalness”) are strong determiners of a disgust response [10,11]. Moreover, food neophobia, defined as aversion to eating new and unfamiliar food, plays a key role in the acceptance of novel food [12,13]. 

As a consequence, the unfamiliarity with insects as food may represent also a hindrance for consumption and market development, especially in cultures where insects’ consumption is not usual [11]. In particular, neophobia and organoleptic features of edible insects in comparison with the features of other well known food (e.g., meat and legumes) is seen as a decisive obstacle to consumers’ acceptance [9,14,15,16,17]. In order to tackle these issues, previous studies suggested to integrate invisible insects in food preparation and/or to associate them with attractive flavors [7,18]. Furthermore, many authors underlined how the food product preparation affects the willingness to eat insects [9,11,19]. For instance, adding insects to familiar preparations (e.g., bread or pasta) or incorporating minced or powdered insects into ready-to-eat preparations, seemed to effectively increase the liking and willingness to try this kind of food in comparison to adding visible insects to meals or proposing them in their ‘‘whole form” [20,21,22,23]. Other authors highlight how consumers may show different behaviors towards the quality and presentation of insect-based food according to their own individual features and socio-cultural background [11,20,24,25], and also in relation to their risk-perceptions in terms of worries for health and production technologies [26,27]. Therefore, it is clear that consumer acceptance of insect-based food may depend on the amount, quality and source of information they receive and provide [26,28,29]. 

Moving the focus from research to policymaking, in many European countries, the sale of insects for human consumption is still illegal, even though the EU Commission is developing regulatory frameworks, environmental and quality standards to prevent risks for consumers from the consumption of novel food [30]. Specifically, the EU “Novel Food Regulation no. 2015/2283”, in effect since 1st January 2018, allows to request the authorization for the commercialization of novel food [6] and the European Food Safety Authority (EFSA) is working on an evaluation of the risk profile related to the production and consumption of insects as food and feed [31]. However, only some EU countries (e.g., Belgium, Netherlands, Denmark, Finland and Germany) have adopted their own internal regulations for the trading of insect-based food, and this affects the spread of retailers selling insect products and their availability on the market, and at the same time, may cause concerns in consumers about safety and healthiness of these food products. 

In the light of this complex scenario, the use of a comprehensive consumer-oriented approach is crucial to simultaneously analyze the factors influencing entomophagy and thus to provide overall insights for its diffusion [6,32]. Therefore, this research aims to advance the knowledge on entomophagy by supplying information to improve consumer acceptance in Italy. This is done by carrying out the characterization of a sample of consumers according to their willingness to taste several types of insect-based food and by taking into account the direct connections among the consumers’ features. 

Thus, a multiple criteria decision aiding (MCDA) approach is applied here, starting from the data gathered from direct consumer questionnaires. MCDA is an umbrella term describing a collection of formal approaches, which take into account multiple criteria in helping individuals or groups to explore decisions that matter [33]. A decision can be tackled through MCDA when there are different choices or alternatives to be judged as more desirable than others by means of criteria that may be in conflict to a substantial extent [34]. Specifically, the applied MCDA approach is called the dominance-based rough set approach (DRSA) because it is based on the rough sets theory and seeks to characterize the groups of consumers by means of simple “*If … then …*” decision rules [35].

Indeed, the decision rules inform about the relationships between conditions and decisions; in this way, the rules enable traceability of the decision support process and give understandable justifications for the decision to be made, so that the resulting preference model constitutes a ‘glass box’ [36]. Then, DRSA has been successfully applied in a variety of fields such as medical diagnosis, engineering reliability, empirical studies of material data, airline market and evaluation of bankruptcy risk [36,37]. On the other hand, applications on food science with focus on consumer analysis are still scarce [38,39]. Moreover, despite a growing interest towards entomophagy both by civil society and scholars, this topic is rather unexplored, showing a knowledge gap between curiosity-driven tasting and actual acceptance [20], which should be filled by applying discovering approaches [40]. For all these reasons, DRSA is considered suitable to explore the topic of entomophagy and to provide in depth analysis of consumers’ attitude towards insect-based food. 

The paper is organized as follows. After describing the methodology for the data collection, the DRSA is illustrated both from the theoretical and the empirical perspectives (Section 2). Then, Section 3 shows the results of the descriptive statistics of the sample and the DRSA application. Section 4 provides a detailed discussion of the results with a focus on those obtained from DRSA. Finally, the concluding remarks are reported in Section 5.

## 2. Materials and Methods 

### 2.1. Data Collection

A questionnaire for the evaluation of the willingness to taste insect-based food was managed via “Google forms” in September 2019. The sample inclusion criteria were: being Italian, ≥18 years old and not being vegetarian or vegan; the sample consisted of 310 Italian consumers (61.6% female and 38.4% male) with age ranging from 18 to 81 years old. The questionnaire was supplied together with the definition of entomophagy, a brief overview of the environmental and nutritional benefits of edible insects, and a summary of the EU “Novel Food Regulation no. 2015/2283”. Moreover, the questionnaire consisted of 18 questions and was structured into the following 4 sections.

- **Section 1—Willingness to taste insect-based food.** This section aimed to investigate the attitude towards the consumption of insects by humans and by cattle, pigs and chickens as feed. Therefore, four different kinds of food, in terms of the degree of processing and perception of the insects as an ingredient, were presented as pictures in the online survey: (1) meat, fish, eggs and milk obtained from animals raised with insect-based feed; (2) protein food supplements based on insect flour (e.g., cricket flour); (3) cookies made from wheat and insect flour; (4) cookies containing visible insects. Consumers were asked to choose which product they were more willing to taste, or else to declare that they were not willing to taste insect-based food in any form or preparation.
- **Section 2—Socio-demographic information and consumers’ habits.** The second section looked to obtain specific consumer information regarding gender, age, monthly income, habitual travel outside Europe [16,41], sports activity [2], raw seafood consumption [42] and insect-based food knowledge [16,18]. Moreover, consumers’ care for food nutritional and environmental aspects were investigated, asking them to assign a score (from 1 = irrelevant to 3 = determinant) to assess how nutritional and environmental features determine their food choices [43,44].
- **Section 3—Consumers’ attitude towards novel food and innovative technologies for food preparation.** In this section, consumers’ neophobia and trust towards innovative technologies for food preparation were assessed, because these individual features have been indicated as important predictors of the acceptance of insects as food [1,16,45,46]. Hence, consumers were asked to assign a score from 1 (I do not trust novel food not even if I know what it contains/The innovative technologies for food preparation are useless and can be harmful) to 4 (I am always looking for novel and different food, I taste everything /The innovative technologies can be fundamental to produce nourishing and sustainable food) in order to investigate their approach towards the consumption of novel food as well as with respect to the proposed innovative technologies.
- **Section 4—Factors affecting the willingness to taste insect-based food.** This last section aimed to evaluate the role of specific insect-based food characteristics in determining consumers’ acceptance. Therefore, consumers were asked to assign the importance to a food safety certification and to a pleasant taste, smell and consistency by expressing a score from 1 (Irrelevant) to 4 (Crucial). In addition, the role of attending a “cooking show” or a “bug banquet” held by a well-known chef [47] was also considered an important factor, so the consumers were asked to assign a score from 1 (No, I would not taste anyway) to 3 (A presentation held by a well-known chef would be relevant for my acceptance).

### 2.2. The Dominance-Based Rough Set Approach

DRSA is a multiple criteria decision aiding (MCDA) method developed by Greco et al. [48,49] and it is an extension of the classical rough set approach (CRSA) [50,51], because it takes into account the preferences of a decision-maker and the typical inconsistency of decision problems [52]. Hence, DRSA substitutes the indiscernibility relation with a dominance relation in the rough approximation of decision classes, making it possible to discover the inconsistencies with respect to the dominance principle [53]. In other words, DRSA is a conceptual framework for the discovery of decision rules that have a syntax concordant with the dominance principle [48,54]. 

DRSA is applied by following the stepwise procedure described by Błaszczyński et al. [55,56] and reported hereafter.

- **Decision table**: Let us consider a decision table including a finite universe of objects U evaluated on a finite set of condition attributes $F=\left\{f_{1},\;\ldots ,\;f_{n}\right\}$ and on a single decision attribute  d. The set of the indices of attributes is denoted by I={1, …,n}. Without loss of generality, $f_{i}\;:\;U\to \;ℜ$ for each i=1, …, n, and, for all objects $x,\;y\;\in U,\;f_{i}\left(x\right)\geq f_{i}\left(y\right)$ means that “x is at least as good as y with respect to attribute i”, that is denoted by $x\;\geq_{i}\;y$. Therefore, it is supposed that $\geq_{i}\;y$ is a complete preorder defined on U based on the quantitative and qualitative evaluations $f_{1}\left(\cdot \right)$. Moreover, the decision attribute d makes a partition of U into a finite number of decision classes $Cl=\left\{Cl_{1},\;\ldots ,\;Cl_{m}\right\}$ such that each x∈U belongs to one and only one class $Cl_{t},\;t=1,\;\ldots ,\;m$. It is assumed that the classes are preference ordered, e.g., for all r, s=1, …, m, such that r>s, the objects from $Cl_{r}$ are preferred respect to the objects from $Cl_{s}$. More precisely, if ≥ is a comprehensive weak preference relation on U, e.g., if for all x, y ∈U, x≥y means that x is at least as good as y, then it is supposed that $\left[x\in Cl_{r},\;y\in Cl_{s},\;r>s\right]\;\Rightarrow x>y$, where x>y means x≥y and not y≥x. These assumptions are typical of an ordinal classification problem with monotonicity constraints, where the decision table includes examples of ordinal classification that represent an input preference information to be analyzed by using DRSA.
  The sets to be approximated are defined as upward union and downward union of decision classes, respectively:
  $$Cl_{t}^{\geq }=\underset{s\geq t}{\cup }Cl_{s},\;Cl_{t}^{\leq }=\underset{s\leq t}{\cup }Cl_{s},\;t=1,\;\ldots ,\;m.\;$$
  The statement $x\in Cl_{t}^{\geq }$ means that x belongs to at least class $Cl_{t}$, while $x\in Cl_{t}^{\leq }$ means that x belongs to at most class $Cl_{t}$. Let us highlight that $Cl_{1}^{\geq }=Cl_{m}^{\leq }=U,\;Cl_{m}^{\geq }=Cl_{m}\;$ and $Cl_{1}^{\leq }=Cl_{1}$. In addition, for t=2, …, m,
  $$Cl_{t-1}^{\leq }=U-Cl_{t}^{\geq }\;\;\mathrm{and}\;Cl_{t}^{\geq }=U-Cl_{t-1}^{\leq }.\;$$
- **Dominance cones**: The approximation of upward and downward unions of decision classes is represented by granules of knowledge that are generated by attributes (i.e., criteria). These granules are also defined as dominance cones in the attribute values space. Specifically, x dominates y with respect to the set of attributes P⊆F (that is x  P−dominates y), denoted by $xD_{P}y$, if for every attribute $f_{i}\in P,\;f_{i}\left(x\right)\geq f_{i}\left(y\right)$. The relation of P-dominance is reflexive and transitive, namely it is a partial preorder.
  Given a set of attributes P⊆I and x∈U, the granules of knowledge used for approximation in DRSA are as follows: a set of objects dominating x, called P-dominating set, given by $D_{P}^{+}\left(x\right)=\left\{y\in U:yD_{P}x\right\}$; a set of objects dominated by x, called P-dominated set, given by $D_{P}^{-}\left(x\right)=\left\{y\in U:xD_{P}y\right\}$.
  In other words, object x dominating object y on all the considered attributes also dominates y on the decision (i.e., the object should be assigned to at least as good decision class as y). The objects that satisfy the dominance principle are consistent, while those violating the dominance principle are called inconsistent.
- **Approximation of ordered decision classes**: The P-lower approximation of $Cl_{t}^{\geq }$ denoted by $\underline{P}\left(Cl_{t}^{\geq }\right)$, and the P-upper approximation of $Cl_{t}^{\geq }$, denoted by $\bar{P}\left(Cl_{t}^{\geq }\right)$, are defined as follows (t=2, …, m):
  $$\underline{P}\left(Cl_{t}^{\geq }\right)=\left\{x\in U:D_{P}^{+}\left(x\right)\subseteq Cl_{t}^{\geq }\right\}\;\bar{P}\left(Cl_{t}^{\geq }\right)=\left\{x\in U:D_{P}^{-}\left(x\right)\cap Cl_{t}^{\geq }\neq \emptyset \right\}.$$
  In the same way, one can define the P-lower approximation and P-upper approximation of $Cl_{t}^{\leq }$ as follows (t=1, …, m−1):
  $$\underline{P}\left(Cl_{t}^{\leq }\right)=\left\{x\in U:D_{P}^{-}\left(x\right)\subseteq Cl_{t}^{\leq }\right\},\;\bar{P}\left(Cl_{t}^{\leq }\right)=\left\{x\in U:D_{P}^{+}\left(x\right)\cap Cl_{t}^{\leq }\neq \emptyset \right\}.$$
  The P-lower approximation and P-upper approximation satisfy the following inclusion property, for all P⊆F:
  $$\underline{P}\left(Cl_{t}^{\geq }\right)\subseteq \;Cl_{t}^{\geq }\subseteq \bar{P}\left(Cl_{t}^{\geq }\right),\;t=2,\;\ldots ,\;m,\;\underline{P}\left(Cl_{t}^{\leq }\right)\subseteq \;Cl_{t}^{\leq }\subseteq \bar{P}\left(Cl_{t}^{\leq }\right),\;t=1,\;\ldots ,\;m-1.$$
  The P-lower approximation and P-upper approximation of $Cl_{t}^{\geq }$ and $Cl_{t}^{\leq }$ hold the complementarity property, according to which:
  $$\underline{P}\left(Cl_{t}^{\geq }\right)=U-\bar{P}\left(Cl_{t-1}^{\leq }\right)\;\mathrm{and}\;\bar{P}\left(Cl_{t}^{\geq }\right)=U-\underline{P}\left(Cl_{t-1}^{\leq }\right),\;t=2,\;\ldots ,\;m,\;\underline{P}\left(Cl_{t}^{\leq }\right)=U-\bar{P}\left(Cl_{t+1}^{\geq }\right)\;\mathrm{and}\;\bar{P}\left(Cl_{t}^{\leq }\right)=U-\underline{P}\left(Cl_{t+1}^{\geq }\right),\;t=1,\;\ldots ,\;m-1.$$
  The P-boundary of $Cl_{t}^{\geq }$ and $Cl_{t}^{\leq }$ denoted by $Bn_{P}\left(Cl_{t}^{\geq }\right)$ and $Bn_{P}\left(Cl_{t}^{\leq }\right)$, respectively, are defined as follows:
  $$Bn_{P}\left(Cl_{t}^{\geq }\right)=\bar{P}\left(Cl_{t}^{\geq }\right)-\underline{P}\left(Cl_{t}^{\geq }\right),\;t=2,\;\ldots ,\;m,\;Bn_{P}\left(Cl_{t}^{\leq }\right)=\bar{P}\left(Cl_{t}^{\leq }\right)-\underline{P}\left(Cl_{t}^{\leq }\right),\;t=1,\;\ldots ,\;m-1.$$
  Due to this complementarity property, $Bn_{P}\left(Cl_{t}^{\geq }\right)=Bn_{P}\left(Cl_{t-1}^{\leq }\right)$, for t=2, …, m.
- **Quality of approximation:** For every P⊆F, the quality of approximation of the ordinal classification Cl by a set of attributes P is defined as the ratio of the number of objects P-consistent with the dominance principle and the number of all the objects in U. Since the P-consistent objects are those which do not belong to any P-boundary $Bn_{P}\left(Cl_{t}^{\geq }\right),\;t=2,\;\ldots ,\;m$, or $Bn_{P}\left(Cl_{t}^{\leq }\right),\;t=1,\;\ldots ,\;m-1$, the quality of approximation of the ordinal classification Cl by a set of attributes P, can be written as follows:
  $$\gamma_{P}\left(Cl\right)=\frac{\left|U-\left(\underset{t=2,\;\ldots ,\;m}{\cup }Bn_{P}\left(Cl_{t}^{\geq }\right)\right)\right|}{\left|U\right|}=\frac{\left|U-\left(\underset{t=1,\;\ldots ,\;m-1}{\cup }Bn_{P}\left(Cl_{t}^{\leq }\right)\right)\right|}{\left|U\right|},$$
  $\gamma_{P}\left(Cl\right)$ is seen as a degree of consistency of the objects from U, where P is the set of attributes (i.e., criteria) and Cl is the considered ordinal classification. Furthermore, for every P⊆F, the accuracy of approximation of union of ordered classes $Cl_{t}^{\geq },\;Cl_{t}^{\leq }$ by a set of attributes P is defined as the ratio of the number of objects belonging to P-lower approximation and to P-upper approximation of the union. The accuracy of approximation $\alpha_{P}\left(Cl_{t}^{\geq }\right)$, $\alpha_{P}\left(Cl_{t}^{\leq }\right)$ can be written as follows:
  $$\alpha_{P}\left(Cl_{t}^{\geq }\right)=\frac{\left|\underline{P}\left(Cl_{t}^{\geq }\right)\right|}{\left|\bar{P}\left(Cl_{t}^{\geq }\right)\right|},\;\alpha_{P}\left(Cl_{t}^{\leq }\right)=\frac{\left|\underline{P}\left(Cl_{t}^{\leq }\right)\right|}{\left|\bar{P}\left(Cl_{t}^{\leq }\right)\right|}\;.\;$$
- **Reduction of attributes**: Each minimal subset P⊆F such that $\gamma_{P}\left(Cl\right)=\gamma_{F}\left(Cl\right)$ is called reduct of Cl, and it is denoted by $RED_{Cl}$. There are more than one reduct for a given set of U. Indeed, the intersection of all reducts is defined the core and it is denoted by $CORE_{Cl}$. The attributes in this core cannot be removed without affecting the quality of approximation. Therefore, in set F, there are the following three categories of attributes: (i) indispensable attributes included in the core; (ii) exchangeable attributes included in some reducts but not in the core; (iii) redundant attributes, neither indispensable neither exchangeable, which are not included in any reduct.
- **Decision rules**: The approximations of upward and downward unions lead to a generalized description of objects in terms of “*if …, then …*” decision rules. For a given upward or downward union of classes, $Cl_{t}^{\geq }$ or $Cl_{s}^{\leq }$, the decision rules induced under a hypothesis that objects belonging to $\underline{P}\left(Cl_{t}^{\geq }\right)$ or $\underline{P}\left(Cl_{s}^{\leq }\right)$ are positive examples, and all the others are negative, suggest a certain assignment to class $Cl_{t}$ or better, or to class $Cl_{s}$ or worse, respectively. Nevertheless, the decision rules induced under a hypothesis that objects belonging to $\bar{P}\left(Cl_{t}^{\geq }\right)$ or $\bar{P}\left(Cl_{s}^{\leq }\right)$ are positive examples, and all the others are negative, suggest a possible assignment respectively to class $Cl_{t}$ or better, or to class $Cl_{s}$ or worse. Finally, the decision rules induced under a hypothesis that objects belonging to the intersection $\bar{P}\left(Cl_{s}^{\leq }\right)\cap \bar{P}\left(Cl_{t}^{\geq }\right)$ are positive examples, and all the others are negative, suggest an approximate assignment to some classes between $Cl_{s}$ and $Cl_{t}$ (s<t).
  In the case of preference ordered description of objects, the set U is composed of examples of ordinal classification. Then, the following five types of decision rules are considered:
  (1) Certain $D_{\geq }$ decision rules, which provide the lower profile descriptions for objects belonging to $\underline{P}\left(Cl_{t}^{\geq }\right)$: if $f_{i1}\left(x\right)\geq r_{i1}$ and … and $f_{ip}\left(x\right)\geq r_{ip}$, then $x\in Cl_{t}^{\geq }$, $\left\{i_{1,\;\ldots ,\;}i_{p}\right\}\subseteq I,\;t=2,\;\ldots ,\;m,\;r_{i1},\;\ldots ,\;r_{ip}\in ℜ$;
  (2) Possible $D_{\geq }$ decision rules, which provide the lower profile descriptions for objects belonging to $\bar{P}\left(Cl_{t}^{\geq }\right)$: if $f_{i1}\left(x\right)\geq r_{i1}$ and … and $f_{ip}\left(x\right)\geq r_{ip}$, then $x\;possibly\;belongs\;to\;Cl_{t}^{\geq }$,
    $\left\{i_{1,\;\ldots ,\;}i_{p}\right\}\subseteq I,\;t=2,\;\ldots ,\;m,\;r_{i1},\;\ldots ,\;r_{ip}\in ℜ$;
  (3) Certain $D_{\leq }$ decision rules, which provide the upper profile descriptions for objects belonging to $\underline{P}\left(Cl_{t}^{\leq }\right)$: if $f_{i1}\left(x\right)\leq r_{i1}$ and … and $f_{ip}\left(x\right)\leq r_{ip}$, then $x\in Cl_{t}^{\leq }$,
    $\left\{i_{1,\;\ldots ,\;}i_{p}\right\}\subseteq I,\;t=1,\;\ldots ,\;m-1,\;r_{i1},\;\ldots ,\;r_{ip}\in ℜ$;
  (4) Possible $D_{\leq }$ decision rules, which provide the upper profile descriptions for objects belonging to $\bar{P}\left(Cl_{t}^{\leq }\right)$: if $f_{i1}\left(x\right)\leq r_{i1}$ and … and $f_{ip}\left(x\right)\leq r_{ip}$, then $x\;possibly\;belongs\;to\;Cl_{t}^{\leq }$,
    $\left\{i_{1,\;\ldots ,\;}i_{p}\right\}\subseteq I,\;t=1,\;\ldots ,\;m-1,\;r_{i1},\;\ldots ,\;r_{ip}\in ℜ$;
  (5) Approximate $D_{\geq \leq }$ decision rules, which provide simultaneously lower and upper profile descriptions for objects belonging to $Cl_{s}\cup Cl_{s+1}\cup \ldots \cup Cl_{t}$, without possibility of discerning to which class:
    if $f_{i1}\left(x\right)\geq r_{i1}$ and … and $f_{ik}\left(x\right)\geq r_{ik}$ and $f_{ik+1}\left(x\right)\leq r_{ik+1}$ and … and $f_{ip}\left(x\right)\leq r_{ip}$, then $x\in Cl_{s}\cup Cl_{s+1}\cup \ldots \cup Cl_{t}$,
    $\left\{i_{1,\;\ldots ,\;}i_{p}\right\}\subseteq I,\;s,\;t\in \left\{1,\ldots ,m\right\}\;s<t,\;r_{i1},\;\ldots ,\;r_{ip}\in ℜ$.
  The first and the third types of rule represent the certain knowledge extracted from data, while the second and the fourth types of rule represent the possible knowledge. The fifth type of rule represents doubtful knowledge, because it is supported only by inconsistent objects [52,57]. Furthermore, a set of decision rules is complete when it covers all the considered objects (i.e., the examples of ordinal classification) in such a way that the consistent objects are re-assigned to their original classes, while the inconsistent objects are assigned to clusters of classes referring to this inconsistency. A set of decision rules is minimal when it is complete and non-redundant [58].

### 2.3. DRSA Empirical Model

DRSA is applied in this research, because it shows the following advantages [36,59]: (i) it connects directly the choice to the condition attributes that determine it (i.e., the type of insect-based food as a decision attribute with the consumers’ features); (ii) it links the condition attributes to the decision attribute through a GAIN-type preference information and a COST-type preference information (i.e., the higher the education level the higher the willingness to taste insect-based food, or the lower the age the higher the willingness to taste insect-based food); (iii) it analyzes the decision rules to identify the object (i.e., consumer) supporting the choice and that justify it; (iv) it deals with both quantitative and qualitative data, and also with the inconsistencies that do not need to be removed before the analysis; (v) it acquires a posteriori information (i.e., consumer groups) about the most relevant attributes that delineate the objects in the form “*if ..., then ...*” decision rules, which are easy to interpret.

Table 1 shows the DRSA input information. More specifically, the names and codes of the criteria as well as the scale of measurement are based on the questionnaire, while the preference for each criterion (GAIN or COST) was identified in accordance with the relevant scientific literature. It should be recalled that GAIN means that the greater the value of a criterion, the greater the preference, while COST means that the lower the value of a criterion, the greater the preference [48,49]. 

This input information, together with the data gathered from the questionnaires, was elaborated using the software “jMAF” developed by the Laboratory of Intelligent Decision Support Systems (Poznań University of Technology, Poland) [55,56]. The extraction of the minimal set of certain decision rules (i.e., the first and the third types of rule) was carried out by applying the Dominance-based Learning from Examples Module (DOMLEM) algorithm [48,60] already implemented in the software. The subsequent characterization of the consumer groups was carried by interpreting the decision rules after the evaluation of their performance through the reclassification of the inconsistent objects [36]. 

**Table 1:** List of criteria with associated codes, scale of measurement and preference information according to the relevant scientific literature.

Criterion and Code	Scale of Measurement	Preference	Reference
SECTION 1: Willingness to taste insect-based food			
Food (FOOD)	(1, 2, 3, 4, 5)	GAIN (decision field)	[2,20,29]
SECTION 2: Socio-demographic information and consumers’ habits			
Gender (GENDER)	(0, 1)	COST	[16,18]
Age (AGE)	(Continuous)	COST	[16,61]
Education (EDU)	(1, 2, 3)	GAIN	[61,62]
Income (INC)	(1, 2, 3, 4)	COST	[5,63]
Sport (SPORT)	(1, 2, 3, 4)	GAIN	[2]
Travel (TRAV)	(1, 2, 3)	GAIN	[11]
Knowledge (KNOW)	(1, 2, 3)	GAIN	[16,18]
Raw seafood (SEAF)	(1, 2, 3)	GAIN	[42]
Nutrition (NUT)	(1, 2, 3)	GAIN	[2,16]
Environment (ENV)	(1, 2, 3)	GAIN	[6,16]
SECTION 3: Consumers’ attitude towards novel food and innovative technologies for food preparation			
Novel food (NEO)	(1, 2, 3, 4)	GAIN	[16,46]
Technology (TECH)	(1, 2, 3, 4)	GAIN	[16]
SECTION 4: Intrinsic and extrinsic factors affecting the willingness to taste insect-based food			
Chef (CHEF)	(1, 2, 3)	GAIN	[11]
Taste (TASTE)	(1, 2, 3, 4)	GAIN	[18,40]
Smell (SMELL)	(1, 2, 3, 4)	GAIN	[20,40]
Consistency (CONS)	(1, 2, 3, 4)	GAIN	[20]
Certification (CERT)	(1, 2, 3)	GAIN	[64]

## 3. Results

### 3.1. Descriptive Statistics of the Sample

The mean age of respondents was 33 years old (S.D. = 10.2 years). More than a half of them hold a high school degree and a third hold a university degree, showing a slight bias towards higher education levels. This bias may be attributed to the use of the electronic form to carry out the survey. The descriptive statistics of the sample are summarized in Table 2.

Table 3 summarizes the frequencies concerning the consumers’ attitude towards novel food and innovative technologies for food preparation, and frequencies of other factors affecting the willingness to taste insect-based food. Regarding the consumers’ food choices, the answers showed that most of the participants in the survey (50.6%) were willing to taste meat, fish, eggs or milk made from animals raised with insect-based feed. Furthermore, 22.9% of consumers were not willing to taste insect-based food in any form of preparation; 16.8% declared a willingness to taste cookies made from wheat and insect flour with invisible insects; 7.4% of consumers were willing to take protein food supplements based on insect flour, such as cricket flour; and only 2.3% agreed to taste cookies containing visible insects. 

On the other hand, the results of the survey showed a general positive attitude towards novel food. Indeed, almost half of consumers (44.5%) declared to be glad to taste novel food if its appearance and smell are attractive. Moreover, less than half of them (37.7%) declared to sometimes taste novel food, but only if they are well informed about the food’s characteristics, while 10% of the sample is always looking for novel and different food and hence, taste everything. A limited share of respondents (7.8%) do not trust novel food, and was not willing to taste even if well informed about its characteristics or ingredients.

Innovative technologies for food preparation appeared to be fundamental in obtaining nourishing and sustainable food according to 46.1% of consumers, while 44.9% of respondents believed that the benefits of these technologies are often overrated, can reduce the natural quality of food and there is already plenty tasty and nourishing food on the market. A limited share of consumers (9%) were against innovative food technologies, because they are considered useless and harmful.

The presence of a food safety certification was perceived as reassuring for 42.9% of consumers, while 30.6% of them declared that certification does not improve their current acceptance of insect-based food and 26.5% of consumers would not taste anyway. 

The majority of respondents considered intrinsic food features (taste, smell, consistency) crucial in their willingness to taste insect-based food (Table 4). Moreover, about 30% of the sample declared not to be willing to taste any kind of this food although it can be organoleptically pleasant. In addition, smell was rated as important but not fundamental according to almost a quarter of respondents, while less than 10% of respondents deemed that consistency is not very important. 

Finally, attending an insect-based food event (e.g., cooking show or bug banquet) held by a famous chef was considered a boost for tasting this food by 23.2% of those interviewed, while 35.2% of them would not taste it anyway and 41.6% of them thought that these events are irrelevant in improving insect-based foods acceptance. 

### 3.2. Characterization of the Consumer Groups 

The application of DRSA enabled to obtain the minimal set of 206 certain decision rules that are organized as follows according to the four insect-based foods plus the not-eat option. In particular, 25 decision rules classified the consumers as not willing to taste insect-based food in any form or preparation (i.e., “*at most FOOD 1*” rules); 81 rules classified the consumers willing to taste meat, fish, eggs or milk from animals raised with insect-based feed (i.e., “*at least FOOD 2*”and “*at most FOOD 2*” rules); 57 rules classified the consumers willing to taste protein food supplements based on insect flour (i.e., “*at least FOOD 3*” and “*at most FOOD 3*” rules); 38 rules classified the consumers willing to taste cookies made from wheat and insect flour (i.e., “*at least FOOD 4*” and “*at most FOOD 4*”); and 5 rules classified the consumers willing to taste cookies with visible insects (i.e., “*at least FOOD 5*”).

Table 5 shows, as an example, 8 certain decision rules classifying the consumers into the 5 food classes. The interpretation of the rules follows a general structure where the criteria and their values (i.e., the consumers’ features) are in the first part of the rule (*If…*), while a certain insect-based food class is reported in the second part of the rule (*then…*). For instance, rule no. 82 regarding the class “Food 1” should be interpreted as follows: “*IF consumers are at least 36 years old with an education level between compulsory and high school, they have never heard of insect-based food and are not willing to taste this kind of food although it can be organoleptically pleasant, THEN they are not willing to taste insect-based food in any form or preparation*”. In the same way, rule no. 4 concerning the class “Food 5” should be interpreted as follows: “*IF the consumers are male between 18 and 22 years old with an income up to 1000 € per month, they think that taking part* *in a tasting session held by a well-known chef may strongly improve their willingness to eat insect-based food, and they also give great importance to taste in their food choices, THEN they would be willing to taste cookies with visible insects*”. 

In order to know if the rules can be used to characterize the groups of consumers, the analysis of the rules’ performances was carried out through the reclassification of the consumers for which these rules were induced [56]. The results of this reclassification are summarized as follows. Firstly, the confusion (or misclassification) matrix (Table 6) defines both the consistent objects that were reassigned to their original decision classes and the inconsistent objects that were reclassified into the decision classes referring to this inconsistency. In other words, the confusion matrix identifies the consumers who gave consistent answers and thus actually belong to the declared food class, and the consumers that need to be reclassified in a different food class since they gave incorrect answers with respect to the declared food class. 

This matrix shows that 232 consumers (74.8% of respondents) provided consistent answers and thus are reassigned to the food class declared in the questionnaire; these consumers can be found along the diagonal of the matrix. On the other hand, 21 consumers (6.8% of respondents) represent the incorrect cases that are reassigned to different decision classes. In particular, 19 consumers who declared that they are not willing to taste insect-based food in any form or preparation (class “Food 1”) have the same characteristics as consumers belonging to other food classes. Indeed, 18 consumers are actually willing to taste meat, fish, eggs or milk from animals raised with insect-based feed (class “Food 2”), while one consumer might be willing to taste protein food supplements based on insect flour (class “Food 3”). In addition, two consumers who were willing to taste cookies with visible insects (class “Food 5”) have the same features as those consumers willing to taste meat, fish, eggs or milk from animals raised with insect-based feed (class “Food 2”). 

The confusion matrix does not include the ambiguous cases, which represent the inconsistent consumers that can neither be reassigned to their original decision class nor reclassified. When DRSA is applied to large real-life data sets, ambiguous cases may occur due to significant differences between lower and upper approximations of the unions of decision classes and also to weak decision rules (i.e., rules supported by few objects from lower approximations) [36,56]. These cases are shown in a separated table providing their distribution within the different food classes (Table 7).

Table 7 shows that there are 57 unclassified consumers (18.4% of respondents). In particular, 28 consumers initially declared to be willing to taste meat, fish, eggs or milk from animals raised with insect-based feed, but 6 of them would also actually taste protein food supplements based on insect flour, and likewise 22 consumers would also taste cookies made from wheat and insect flour. Moreover, among the 10 consumers at first willing to taste protein food supplements based on insect flour, there are 8 consumers that would also taste eggs or milk from animals raised with insect-based feed, while one consumer is willing to taste both this last type of food and also cookies made from wheat and insect flour. Within this third food class, there is another consumer that is actually willing to taste the cookies together with the food declared in the questionnaire. Finally, 19 consumers were initially willing to taste cookies made from wheat and insect flour, but actually 18 of them would also taste meat, fish, eggs or milk from animals raised with insect-based feed, while one consumer is also willing to taste protein food supplements based on insect flour. 

The reclassification of the consumers based on the extracted decision rules confirmed their validity to characterize the groups of consumers with the exclusion of the ambiguous cases reported in Table 7. Therefore, the interpretation of the minimal set of certain decision rules enabled to characterize 5 groups of consumers according to the most relevant features displayed by the rules and described hereafter.

- **Group 1—Consumers not willing to eat insect-based food in any form or preparation.** The first consumer group consists of 17 males and 35 females between 35 and 50 years old, their education is mostly at the secondary high school level and the monthly income is over 2100 €. These consumers practice sports occasionally, they have partial knowledge of insect-based food and they never eat raw seafood. Moreover, they are willing to taste new types of food only if detailed information is supplied, and they also think that innovative technologies are not useful for preparing food, because they can be harmful and useless. Finally, this group of consumers is not very interested in the food nutritional aspects and a good smell does not encourage tasting.
- **Group 2—Consumers willing to eat meat, fish, eggs or milk from animals raised with insect-based feed.** The second consumer group consists of 42 males and 107 females, between 26 and 32 years old with a medium-high education level (high school or university degree), and their income per month is between 1100 and 3000 €. About half of them have heard of insect-based food although they do not know what it means, while the remaining consumers have sufficient knowledge and they occasionally eat raw seafood and are thus glad to taste or eat novel food if the smell and consistency are attractive. Moreover, a certification, a chef’s presentation (e.g., cooking show and bug banquet) and a good smell may encourage tasting.
- **Group 3—Consumers willing to eat protein food supplements based on insect flour (i.e., cricket flour).** Consumers of the third group are 7 males and 7 females between 20 and 34 years old, their monthly income is between 2100 and 3000 €. They travel less than once a year, and they are willing to taste novel food only if well informed about its features. These consumers do not trust new technologies for food preparation and they are also doubtful about certification as a tool to encourage tasting and eating insect-based food. In addition, a good taste is considered important but not fundamental in their choices.
- **Group 4—Consumers willing to eat cookies made from wheat and insect flour.** There are 17 males and 16 females between 21 and 25 years old, their monthly income is between 1100 and 2000 €; they travel less than once a year, and occasionally practice sports. Moreover, these consumers like to taste novel food if its appearance and smell are attractive, and they think that innovative technologies for food preparation are overrated and reduce food quality. Finally, they are not encouraged by a chef’s presentation of insect-based food (e.g., cooking show and bug banquet), even though they think that taste is important in determining their choice.
- **Group 5—Consumers willing to eat cookies with visible insects.** There are 3 males and 2 females under 23 years old, their hold a university degree, their income is mostly under 2000 € per month, and they regularly practice a sport. Furthermore, these consumers are always looking for novel and different food and thus are willing to taste everything. Consequently, they believe that innovative technologies are fundamental to produce nourishing and sustainable food. In addition, they think that taking part in presentations held by a well-known chef (e.g., cooking show and bug banquet) strongly improves their own willingness to taste insect-based food.

## 4. Discussion

The main finding of the survey still confirms the low level of acceptance of insect-based food in Italy [13,61,65], because about 23% of respondents declared to be not willing to taste any kind of this food. Moreover, most of the participants in the survey expressed their acceptance of the introduction of insects into their diet only as feed or food supplement, in a form as different as possible from visible insects. In addition, less than 20% of interviewed consumers declared to be available to taste familiar food containing processed insects like cookies made with insect flour or with visible insects. 

The analysis of the consumer groups obtained from the decision rules shows that the consumers strongly rejecting insect-based food in any form or preparation (i.e., Group 1) are the oldest, in accordance with other authors highlighting that an increase in age is associated with a decrease in the probability of accepting insects as a foodstuff [5,16]. These consumers hold an intermediate education level and have partial knowledge of insect-based food. In this sense, some studies [6,7,16,66] reported how complete information about the sustainability and environmental perspectives of edible insects, can positively influence the consumers’ attitude towards tasting and consuming insect-based products. In addition, providing consumers with comprehensive information on edible insects may reduce their fear and increase their purchase probability [32]. Moreover, this group of consumers seems more interested in organoleptic rather than health features of food and are not willing to compromise taste for health and environmental benefits [62,67]. These consumers are also very careful in tasting novel food, as they declare to be unfamiliar with eating raw seafood. This may be considered a further cause of scepticism towards similar products in terms of appearance, like insect-based food. Hence, food neophobia has a strong role in influencing the consumers’ attitude towards edible insects [7,9,15,16,19]. On the contrary, the familiarity with raw seafood consumption may represent an encouragement to taste insect-based food, due to the similarity of grasshoppers and cicadas to crustaceans and the resemblance of aquatic insect larvae’s taste to fish served as ‘*ceviche*’ [68].

The largest group of consumers (i.e., Group 2) showed a good level of acceptance of insects as feed for cattle and fish. Although insect-based feed does not yet exist on the EU market due to the lack of a defined regulatory framework, currently these consumers’ positive attitude may bode good market perspectives in compliance with the findings of other scholars [67,69,70]. However, this also confirms that the majority of consumers are clearly not ready to incorporate insects as such into their diets [13]. In this sense, the acceptance of edible insects may be improved by a proper knowledge of this food, as well as by a higher inclination to taste novel food, especially if it is attractive in terms of consistency and smell. Likewise, these consumers are familiar with raw seafood consumption, hence the availability of further information regarding the similarity between insects and crustaceans (e.g., exoskeletons and antennae, their presence both in the aquatic and terrestrial systems), could improve the acceptability of entomophagy in their food habits [19]. Finally, being involved in thematic cooking shows and bug banquets may also strengthen their positive attitude.

The third group of consumers declared to be ready to accept protein food supplements based on insect flour. It should be recalled that, also in this case, the original product is not directly recognizable due to the high level of processing. The consumers belonging to this group have a medium-high monthly income, and this finding is in line with some studies [42,63] highlighting that unprocessed insects are usually consumed by people with medium-low income especially in developing countries, because their production process is cheap and they can afford a food with a high amount of protein, especially in a period of food shortage. However, recent research [27,32] found that the consumption of unprocessed insects is increasing also among high-income consumers, due to their better awareness about the nutritional benefits of this food. Moreover, the consumers in this group are still sceptic about tasting novel food, which explains why they prefer a food in which the insect is completely processed. In addition, they travel more than the consumers of other groups, but this feature still does not positively affect the tasting and consumption of unprocessed insects. Even though a regular tourist may have a strong propensity to look for unknown food and to appreciate the novelty of gastronomic experiences in a foreign environment, sometimes local food consumption is not a priority when the consumption of food is not the main goal of a trip and it is treated as a daily task, where the experience of meals does not increase the curiosity to try new food [71,72]. These consumers do not trust innovative technologies for food preparation, and they believe that a certification cannot encourage the tasting of insect-based food. This scepticism may be due to food safety concerns such as microbiological and chemical hazards especially in some developed countries, where potential consumers are deterred from incorporating or even thinking of including insects in their diets [26,27]. 

The fourth group is willing to eat cookies containing insect flour and includes consumers between 21 and 25 years old with a medium income. These findings are in line with studies showing that young and medium-income consumers are readier to taste and eat insect-based food as they have a better awareness of their nutritional benefits and entomophagy is seen as a thrilling experience [27,32,68]. It should be also noted that this group shows a gender balance, differently from other studies reporting that women are more disgusted by products of animal origin and more reluctant to accept insect-based food [15,61,73]. In this sense, a favorable behavior towards insect-based food both by males and females may be related to the curiosity about the taste and consistency of such a new food, which is typical of young people [19,40,66]. Hence, curiosity and attention towards taste and smell is the most important factor in their acceptance of this specific type of insect-based food [40], as they think that innovative technologies for food production may decrease food quality and the cooking shows or bug banquets do not influence their choices. 

Consumers included in the fifth group are the youngest and hold a university degree. Indeed, a high education level plays a key role in the acceptance of edible insects [61]. Moreover, they practice sports regularly and this finding is in compliance with a study reporting that people who exercise regularly take care of the protein profile of their food, thus they are more positive towards unprocessed insects or insects as a visible ingredient [2].

In addition, these consumers believe that innovative food technologies are of fundamental importance in food production, although their novel food knowledge and involvement in sustainability issue is limited and not directly related to their choices. They are always seeking for new food experiences such as cooking shows and bug banquets, confirming the results of other studies highlighting that entomophagy may take on, especially in young people, the symbolic role of toughness or bravery, hence more related to a thrilling experience rather than to a conscious food choice [16,68].

The overall findings of this research clearly show the low level of acceptance of insect-based food, a very low knowledge of the features of this new food, and in particular, a scarce expertise in certain subjects (e.g., health and environmental benefits) that can encourage its tasting and consumption. Therefore, providing targeted information about entomophagy’s benefits [65] may increase the consumers’ acceptance of insect-based food. Specifically, both the similarity of insects with raw seafood and crustaceans and the general positive nutritional and environmental aspects should be better pointed out. At the same time, supplying people with opportunities to try insects during tasting sessions, special events, food fairs and “bug banquets” held by experts could reassure consumers about the organoleptic characteristics of these food and the social acceptability of entomophagy. In this sense, some studies dealing with the sensory acceptance of insect-enriched food confirmed that if insect-based food is properly presented, consumers may show a surprisingly high acceptance [74,75,76].

Young people currently represent only a niche market, but they can play a role in changing trends as future buyers and consumers. Conversely, for the majority of consumers, the willingness to accept insects in the form of feed stuffs or supplements can represent a first step towards entomophagy, if the positive characteristics of the products are properly communicated. It should be also highlighted that accepting to taste invisible insects may represent a first step towards overcoming the rejection of unfamiliar food and can further increase peoples’ willingness to eat unprocessed insects [9]. As other authors point out [1,64,77], previous positive experiences and repeated consumption facilitate food preferences and willingness to introduce novel items in food habits [78]. With a view on legalizing the trade of these products, it is reasonable to think that food products containing processed insects as ingredients can be more promising than marketing products containing entire insects [1,9]. However, it is also essential to put in place the appropriate strategies to communicate the various positive characteristics of these products.

## 5. Conclusions

This research provides a useful contribution to understand how consumers’ features may affect different behaviors towards entomophagy, since it advances the knowledge on this topic through new and detailed information. The DRSA enabled to inquire the consumers’ attitudes dealing with uncertain data and taking into account the interactions among the individuals’ features, thus providing a clear link between the consumers’ choice and the factors that determine it. Although the decision rules cannot be used to characterize the entire Italian population due to the limited sample, the results can be considered a first step for future broader investigations and can suggest possible educational and communication strategies to improve consumer acceptance. According to the results obtained, future research will be carried out by implementing direct tasting sessions to reduce the uncertainty of data because of the illustration of insect-based food with pictures, and also by applying the variable-consistency dominance-based rough set approach (VC-DRSA) that will be more suitable to deal with a large real-life data set and thus to reduce the number of inconsistent cases. 

## Figures and Tables

**Table 2:** Sample statistics on socio-demographic information and consumers’ habits.

Criterion	Scale of Measurement	Frequency (%) or Mean ± SD
Age	Continuous	32.5 ± 10.2
Gender	0 = Male	38.4%
	1 = Female	61.6%
Education	1 = Compulsory school	14.8%
	2 = High school	54.8%
	3 = University degree or postgraduate	30.4%
Income	1 = Up to 1000 €	20.3%
	2 = From 1100 to 2000 €	45.8%
	3 = From 2100 to 3000 €	18.7%
	4 = More than 3000 €	15.2%
Sport	1 = Never	18.1%
	2 = Occasionally	45.5%
	3 = Regularly (at least twice per week)	30.9%
	4 = Competitive level	5.5%
Travel	1 = Never	32.2%
	2 = Occasionally (less than once per year)	42.6%
	3 = Regularly (at least once per year)	25.2%
Knowledge	1 = I have never heard about insect-based food for human consumption	12.2%
	2 = I have heard about insect-based food, but I do not know what it means	49.4%
	3 = I have heard about insect-based food and I know what it means	38.4%
Raw seafood consumption	1 = Never	32.9%
	2 = Occasionally	57.7%
	3 = Regularly (at least once per week)	9.4%
Care of nutritional aspects in food choice	1 = Irrelevant	5.8%
	2 = Relevant but not determining	51.3%
	3 = Absolutely fundamental	42.9%
Care of environmental aspects in food choice	1 = Irrelevant	10.7%
	2 = Relevant but not determining	59.0%
	3 = Absolutely fundamental	30.3%

**Table 3:** Sample statistics on acceptance of novel food, innovative technologies for food preparation and other factors affecting the willingness to taste insect-based food.

Criterion	Scale of Measurement	Frequency (%)
Type of insect-based food	1 = I am not willing to eat insect-based food in any form or preparation	22.9%
	2 = I am willing to eat meat, fish, eggs or milk made from animals raised with insect-based feed	50.6%
	3 = I am willing to eat protein food supplements based on insect flour	7.4%
	4 = I am willing to eat cookies made from wheat and insect flour	16.8%
	5 = I am willing to eat cookies with visible insects	2.3%
Novel food acceptance	1 = I do not trust novel food, not even if I know what it contains	7.8%
	2 = I sometimes taste novel food, but only if I am well informed about its characteristics	37.7%
	3 = I am glad to taste novel food if its appearance and smell are attractive	44.5%
	4 = I am always looking for novel and different food, I taste everything	10%
Innovative technologies acceptance	1 = The innovative technologies for food preparation are useless and can be harmful	9%
	2 = There is plenty tasty and nourishing food available on the market, so there is no need to use innovative technologies to produce more food	23%
	3 = The benefits of innovative food technologies are often overrated and can reduce the natural quality of food	21.9%
	4 = The innovative technologies can be fundamental to produce nourishing and sustainable food	46.1%
Presence of a food safety certification	1 = No, I would not taste anyway	26.5%
	2 = It would not change so much	30.6%
	3 = Yes, I would taste	42.9%
Participation to a tasting session leaded by a well-known chef	1 = No, I would not taste anyway	35.2%
	2 = It would not change so much	41.6%
	3 = Yes, I would taste	23.2%

**Table 4:** Sample statistics about the importance of organoleptic features of insect-based food.

Criterion	Scale of Measurement	Frequency (%)
Importance of taste when evaluating insect-based food	1 = I would not taste any kind of insect-based food	28.4%
	2 = Not very important	5.5%
	3 = Important but not fundamental	16.4%
	4 = Crucial	49.7%
Importance of smell when evaluating insect-based food	1 = I would not taste any kind of insect-based food	29.4%
	2 = Not very important	4.2%
	3 = Important but not fundamental	25.8%
	4 = Crucial	40.6%
Importance of consistency when evaluating insect-based food	1 = I would not taste any kind of insect-based food	30.0%
	2 = Not very important	7.4%
	3 = Important but not fundamental	22.6%
	4 = Crucial	40.0%

**Table 5:** Examples of certain decision rules for each food class.

Rule no.	Decision Rule	Food Class
82	If (Age ≥ 36) & (Edu ≤ 2) & (Know ≤ 1) & (Smell ≤ 1) then (Food ≤ 1) |CERTAIN, AT_MOST, 1|	Food 1
64	If (Edu ≥ 2) & (Neo ≥ 3) & (Cert ≥ 2) & (Chef ≥ 2) & (Taste ≥ 2) & (Smell ≥ 2) then (Food ≥ 2) |CERTAIN, AT_LEAST, 2|	Food 2
105	If (Seaf ≤ 2) & (Cert ≤ 1) & (Taste ≤ 1) then (Food ≤ 2) |CERTAIN, AT_MOST, 2|	Food 2
29	If (Age ≤ 37) & (Sport ≥ 3) & (Trav ≥ 3) & (Chef ≥ 3) then (Food ≥ 3) |CERTAIN, AT_LEAST, 3|	Food 3
152	If (Cert ≤ 1) & (Taste ≤ 2) then (Food ≤ 3) |CERTAIN, AT_MOST, 3|	Food 3
9	If (Edu ≥ 3) & (Sport ≥ 2) & (Neo ≥ 4) & (Cons ≥ 3) then (Food ≥ 4) |CERTAIN, AT_LEAST, 4|	Food 4
193	If (Neo ≤ 3) & (Taste ≤ 3) then (Food ≤ 4) |CERTAIN, AT_MOST, 4|	Food 4
4	If (Gender ≤ 0) & (Age ≤ 22) & (Inc ≤ 1) & (Chef ≥ 3) & (Taste ≥ 4) then (Food ≥ 5) |CERTAIN, AT_LEAST, 5|	Food 5

**Table 6:** The confusion matrix. Each column represents the objects in a predicted class, while each row represents the objects in an actual class.

Food Class	PREDICTED				
ACTUAL	1	2	3	4	5
**1**	52	18	1	0	0
**2**	0	129	0	0	0
**3**	0	0	13	0	0
**4**	0	0	0	33	0
**5**	0	2	0	0	5

**Table 7:** The ambiguous cases for each food class and their distribution. Each number in brackets identifies an unclassified consumer.

Food Class	No. of Ambiguous Cases	Distribution of Ambiguous Cases in Food Classes		
		<2, 3>	<2, 4>	<3, 4>
**2**	28	6 (30, 89, 136, 138, 210, 278)	22 (5, 21, 27, 56, 68, 70, 79, 80, 84, 86, 108, 123, 129, 163, 169, 190, 200, 213, 235, 252, 270, 282)	
3	10	8 (40, 45, 49, 55, 174, 201, 211, 259)	1 (305)	1 (155)
4	19		18 (2, 33, 61, 88, 142, 146, 184, 215, 249, 262, 264, 274, 276, 284, 285, 287, 307, 309)	1 (106)
